# A case report: Management of carbamazepine intoxication using hemodialysis followed by continuous venovenous hemodialysis

**DOI:** 10.1177/2050313X241229844

**Published:** 2024-02-08

**Authors:** Heshu Abdullah-Koolmees, Vivian CC Mekel, Agnes I Veldkamp, Martine G Caris, Eleonora L Swart

**Affiliations:** 1 Department of Pharmacy and Clinical Pharmacology, Amsterdam University Medical Center, Location Vrije Universiteit Medical Center, Amsterdam, The Netherlands; 2 Division of Pharmacoepidemiology and Clinical Pharmacology, Department of Pharmaceutical Sciences, Utrecht Institute for Pharmaceutical Sciences, Utrecht University, Utrecht, The Netherlands; 3 Division of Laboratories, Pharmacy, and Biomedical Genetics, Department of Clinical Pharmacy, University Medical Center Utrecht, Utrecht, The Netherlands; 4 Department of Clinical Pharmacy, Amstelland Hospital, Amstelveen, The Netherlands; 5 Department of Internal Medicine, Amsterdam University Medical Center, Location Vrije Universiteit Medical Center, Amsterdam, The Netherlands

**Keywords:** Carbamazepine, intoxication, hemodialysis, CVVHD

## Abstract

An 18-year-old woman presented to the emergency department. She had ingested 43 extended-release tablets of carbamazepine 400 mg. Although the patient had high carbamazepine plasma levels and classified as severe intoxication, her clinical symptoms were less severe than expected. With the combination of hemodialysis and continuous venovenous hemodialysis in addition to usual care, including multiple-dose activated charcoal, a fast decrease (within 3 days) in carbamazepine plasma levels to levels in the therapeutic range was achieved. Only one session of hemodialysis was performed because the clinical status of the patient stabilized. In retrospect, the patient did not suffer severe toxicological symptoms from carbamazepine. Therefore, continuous venovenous hemodialysis could have been discontinued earlier. On the other hand, the fast decrease in carbamazepine plasma levels during extracorporeal treatment may have prevented the development of severe or rebound toxicological symptoms. This case report adds evidence to the successful management of carbamazepine intoxication using hemodialysis followed by continuous venovenous hemodialysis.

## Introduction

Carbamazepine (CBZ) is an antiepileptic drug that has been on the market internationally since 1964. Since then, it has also been used as an analgesic in the treatment of trigeminal neuralgia and neuropathic pain syndromes, and as a mood stabilizer for bipolar disorders. CBZ is formulated as either an immediate-release (IR) suspension and tablets or as extended-release (ER) tablets.

While CBZ’s mechanisms of action have not yet been fully clarified, the blocking of presynaptic voltage-gated sodium channels is known to play a role. These channels are involved in producing and propagating action potentials along excitable cells, such as sensory neurons and muscle cells. They respond to small changes in membrane potential by opening their channel and allowing sodium to enter the cell, which causes membrane depolarization. When an action potential arrives at the neural axon, glutamate is released from synaptic vesicles, which then binds to postsynaptic receptors. This causes the postsynaptic membrane to depolarize and initiates a postsynaptic action potential. CBZ inhibits the release of glutamate and consequently the propagation of the action potential in overexcited neurons, which plays a role in seizures, neuropathic pain, or bipolar disorders.^1,2^ Besides the inhibition of glutamate release, CBZ is also known to inhibit *N*-methyl-d-aspartate and adenosine receptors, the conversion of catecholamines, and possibly the release of other excitatory neurotransmitters from presynaptic neurons.^3,4^

Toxicity from CBZ overdose was first described in 1967.^5^ Although CBZ-related fatalities are uncommon, CBZ is involved in a large proportion of life-threatening anticonvulsant poisoning cases. The US Poison Control Centers documented 1257 cases of toxic CBZ exposure in 2020; 318 had a moderate effect, 52 had a major effect, and one led to death.^4,6,7^

The toxic effects of CBZ are characterized by neurological, cardiac, and gastrointestinal (GI) symptoms. These effects are caused by the interaction of CBZ with various receptors and ion channels.^4,6–19^ First, its neurological effects are nystagmus, ataxia, gross intention tremor, dysarthria, agitation, respiratory depression, coma, and seizures. Second, the cardiac effects observed in CBZ poisoning are sinus tachycardia, bradycardia, hypotension, hypertension, atrioventricular block, premature ventricular contractions, ventricular tachycardia, and junctional escape rhythms.^10,13,14^ Lastly, the GI effects include vomiting, ileus, and the development of so-called pharmacobezoars.^8,9^ Pharmacobezoars occur when many tablets are ingested in a short period of time, combined with a lack of fluid for the tablets to dissolve as well as hypomotility of the GI tract caused by the anticholinergic effects of CBZ. In severe cases of toxicity, paradoxical seizures, ventricular arrhythmias, respiratory depression, and cyclic comas are predominant.

## Extracorporeal treatment

In patients with CBZ overdose, multiple-dose activated charcoal (MDAC) increases elimination and improves clinical outcomes. The repeated administration of activated charcoal interrupts the enterohepatic circulation of CBZ. However, the use of MDAC can be limited by decreased bowel motility or concerns over airway protection. In cases of life-threatening symptoms unresponsive to conventional treatment or a contraindication to MDAC, extracorporeal treatment (ECTR) is recommended.^6–19^ This is despite the low quality of available clinical evidence and the high protein binding capacity of CBZ. In a systematic review by Ghannoum et al.,^6^ the Extracorporeal Treatments in Poisoning (EXTRIP) workgroup concluded that ECTR is useful in cases of severe CBZ poisoning.

ECTR is suggested to be effective because in severe CBZ poisoning, the unbound fraction of CBZ is expected to be increased.^15^ Toxic exposure should be confirmed by plasma concentrations before starting ECTR and ECTR should be continued until clinical improvement is apparent or the CBZ plasma concentration is below 10 mg/L.

Various ECTR techniques can be applied, but the EXTRIP workgroup recommends intermittent hemodialysis (HD) as the preferred technique, because of its common availability and lower complication rates and costs.^6^

The EXTRIP workgroup concluded that continuous techniques are inferior to intermittent techniques with markedly lower clearance rates. However, continuous techniques are usually better tolerated hemodynamically and can be considered an alternative (6,16). In addition, continuous techniques are able to mitigate the rebound effects of CBZ due to secondary peaks in plasma concentration (17).

Here, we report a case of intoxication with CBZ in an 18-year-old patient with severe CNS depression. This case indicates that HD followed by continuous venovenous hemodialysis (CVVHD) is an effective option for treating CBZ intoxication. Written informed consent was obtained from the patient(s) verbally and written consent for their anonymized information to be published in this article.

## Case description

An 18-year-old woman presented to the emergency department of the Amsterdam University Medical Center. She was brought in by a family member after they discovered that she had ingested her mother’s CBZ tablets. She had no prescribed drugs and no known allergies. The patient first stated that she had ingested 20–30 ER tablets of CBZ 400 mg; however, she later confessed to ingesting 43 ER tablets of CBZ 400 mg. At presentation, the airway was free, her respiratory rate was 21 breaths/min with SpO_2_ of 97%, her blood pressure was 108/77 mmHg, and her heart rate was 138 beats/min. Her ECG revealed sinus tachycardia and normal intervals. The patient’s initial Glasgow Coma Scale score was 6 (E1M4V1).^14^ Her body temperature was 37°C, and her body weight was 65 kg. Her arms exhibited signs of automutilation.

The patient received activated charcoal approximately 3 h after ingesting CBZ, following national guidelines. The patient purposely vomited approximately half of the activated charcoal quickly after ingestion, but no tablets were detected.^4,16^ Therefore, a second dose of activated charcoal was administered 3 h after the first dose instead of the recommended 6 h.^4,16^ The patient was transferred to the medium-care unit because she became more somnolent with time. There, she received sodium sulfate and macrogol. As she had ingested 265 mg/kg CBZ, she was classified as having severe CBZ intoxication.^6,13^ Eight hours after ingesting CBZ, the patient’s CBZ plasma concentration was 49.6 mg/L. This can be classified as a severe intoxication as shown in Table 1. Pharmacobezoar of carbamazepine was not diagnosed.

**Table 1.** Intoxication classification according to carbamazepine (CBZ) plasma concentrations.^6^

Carbamazepine plasma levels (mg/L)	Intoxication classification
>10	Light
>20	Moderate
>30	Severe

**Figure 1.**
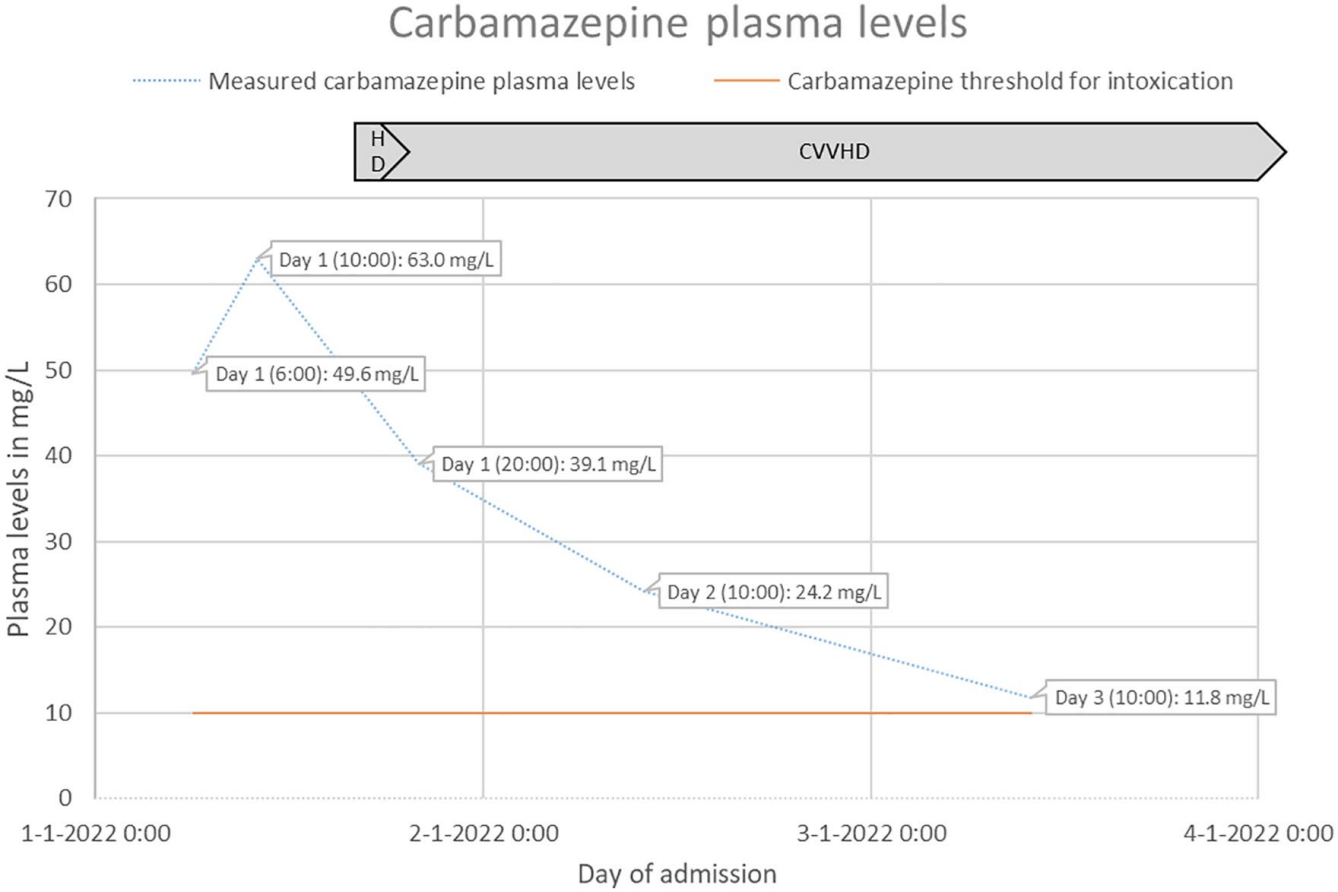
The patient’s measured CBZ plasma levels during hospital admission with a schematic representation of the period of HD and CVVHD. CBZ: carbamazepine; HD: hemodialysis; CVVHD: continuous venovenous hemodialysis.

She was stable until 14 h after ingesting the CBZ; then, she vomited the activated charcoal again, aspirated, and developed respiratory failure, for which mechanical ventilation was initiated in the intensive care unit. Based on her clinical status and an even higher second CBZ plasma concentration of 63 mg/L (Figure 1), HD was started according to national guidelines and EXTRIP recommendations for 3 h, followed by CVVHD to enhance CBZ excretion. Four hours after the start of HD, the patient’s CBZ plasma concentration dropped to 39.1 mg/L (Figure 1).

On the third day of admission, the patient had not developed any cardiac adverse reactions or seizures. Therefore, she was transferred to a secondary care hospital. As her clinical status was stable and her CBZ plasma concentration decreased to 11.8 mg/L (Figure 1) on day 3 of admission, CVVHD was discontinued on day 4 according to national guidelines and EXTRIP recommendations. She was extubated on day 6 but showed elevated liver enzymes. After 9 days, she was awake and alert and transferred to the ward. She suffered from headache and myalgia until day 11. On day 13, her liver laboratory test results improved, and she was discharged from the hospital with follow-up from a psychiatrist. The patient’s measured CBZ plasma levels are shown in Figure 1.

## Discussion

Although the patient had high CBZ plasma levels and classified as severe intoxication, her clinical symptoms were less severe than expected. With the combination of HD and CVVHD in addition to usual care, including MDAC, a fast decrease (within 3 days) in CBZ plasma levels to the therapeutic range was achieved.

At first, HD was initiated to quickly remove a substantial amount of CBZ from the blood. Only one session of HD was performed because the clinical status of the patient stabilized. To extend the excretion of CBZ, the HD session was directly followed by CVVHD.

In retrospect, the patient did not suffer severe toxicological symptoms from CBZ. Therefore, CVVHD could have been discontinued earlier. On the other hand, the fast decrease in CBZ plasma levels during ECTR may have prevented the development of severe or rebound toxicological symptoms. The CVVHD increased the carbamazepine clearance and thereby a possible secondary high carbamazepine concentrations and carbamazepine intoxication symptoms.

In therapeutic use, the absorption of CBZ from the GI tract is relatively slow after the ingestion of ER tablets (*T*_max_ 24 h), faster after the ingestion of IR tablets (*T*_max_ 12 h), and rapidly after the ingestion of a suspension (*T*_max_ 2 h). In toxicological cases, peak plasma concentrations can be prolonged to over 72 h after the ingestion of IR tablets and over 100 h after the ingestion of ER tablets.^4,6^ Crucially, secondary peak plasma concentrations, leading to a rebound of CBZ’s clinical effects, are observed hours after the initial peak.^6^ CBZ plasma concentrations are known to fluctuate during the first 2–3 days due to continued absorption of CBZ from the gut.^4,10^ Various mechanisms contribute to this prolonged and unpredictable absorption pattern: First, ER tablets are designed to dissolve over time; second, CBZ exhibits an enterohepatic cycle; third, when a bolus of CBZ is introduced to the gut, it can cause the aforementioned pharmacobezoars; and lastly, CBZ has intrinsic anticholinergic properties, which can cause hypomotility of the bowel or even ileus, further delaying absorption.^3,4,6,11^

After absorption, CBZ is extensively metabolized by the liver, primarily through cytochrome P450 3A4 (CYP3A4). The primary active metabolite is carbamazepine-10,11-epoxide (CBZE). CBZ and CBZE are approximately 70%–80% and 50% protein bound, respectively, with wide interpatient variability. Protein binding and the ratio of the parent drug to epoxide metabolite are concentration-dependent; when CBZ plasma concentrations are elevated, they result in significantly higher circulating amounts of unbound CBZ and CBZE.^3,12^ Furthermore, CBZ causes autoinduction through epoxidation and hydration in CBZ metabolism. CBZ’s pharmacokinetic is therefore time-dependent and nonlinear.^18,19^ Carbamazepine metabolism may have been elevated through time through autoinduction in this case as well. Autoinduction had a greater impact on carbamazepine metabolism in case of chronic use which was not the case for this patient.

## Conclusion

Overall, more knowledge is required regarding the extent to which HD and CVVHD contribute to the excretion of CBZ and its metabolites from the blood. However, this case report adds evidence to the successful management of CBZ intoxication using HD followed by CVVHD. In this case, the carbamazepine clearance was increased by the CVVHD and probably a secondary high carbamazepine concentrations and carbamazepine intoxication symptoms was prevented.
